# Evaluation of genetic susceptibility to childhood allergy and asthma in an African American urban population

**DOI:** 10.1186/1471-2350-12-25

**Published:** 2011-02-14

**Authors:** Bonnie R Joubert, David M Reif, Stephen W Edwards, Kevin A Leiner, Edward E Hudgens, Peter Egeghy, Jane E Gallagher, Elaine Cohen Hubal

**Affiliations:** 1 US Environmental Protection Agency (EPA), National Exposure Research Laboratory, Durham, NC, USA; 2 EPA, National Center for Computational Toxicology, Durham, NC, USA; 3 EPA, National Health and Environmental Effects Research Laboratory, Durham, NC, USA; 4 Integrated Laboratory Systems, RTP NC, USA; 5 Epidemiology Branch, National Institute of Environmental Health Sciences, National Institutes of Health, Department of Health and Human Services, Research Triangle Park, NC

## Abstract

**Background:**

Asthma and allergy represent complex phenotypes, which disproportionately burden ethnic minorities in the United States. Strong evidence for genomic factors predisposing subjects to asthma/allergy is available.  However, methods to utilize this information to identify high risk groups are variable and replication of genetic associations in African Americans is warranted.

**Methods:**

We evaluated 41 single nucleotide polymorphisms (SNP) and a deletion corresponding to 11 genes demonstrating association with asthma in the literature, for association with asthma, atopy, testing positive for food allergens, eosinophilia, and total serum IgE among 141 African American children living in Detroit, Michigan. Independent SNP and haplotype associations were investigated for association with each trait, and subsequently assessed in concert using a genetic risk score (GRS).

**Results:**

Statistically significant associations with asthma were observed for SNPs in *GSTM1, MS4A2*, and *GSTP1 *genes, after correction for multiple testing. Chromosome 11 haplotype CTACGAGGCC (corresponding to *MS4A2 *rs574700, rs1441586, rs556917, rs502581, rs502419 and *GSTP1 *rs6591256, rs17593068, rs1695, rs1871042, rs947895) was associated with a nearly five-fold increase in the odds of asthma (Odds Ratio (OR) = 4.8, *p *= 0.007). The GRS was significantly associated with a higher odds of asthma (OR = 1.61, 95% Confidence Interval = 1.21, 2.13; *p *= 0.001).

**Conclusions:**

Variation in genes associated with asthma in predominantly non-African ethnic groups contributed to increased odds of asthma in this African American study population. Evaluating all significant variants in concert helped to identify the highest risk subset of this group.

## Background

Asthma is a complex chronic disease of the airway, with notable airflow obstruction, eosinophilic inflammation, bronchoconstriction, and mucus hypersecretion [1]. Asthma and allergy (asthma/allergy) disproportionately burden ethnic minority children in the United States, African Americans in particular [2-4]. Asthmatic individuals of African ancestry have also been characterized with higher IgE levels, higher dependence on treatment, and experience more severe clinical symptoms compared to Whites [2]. Socioeconomic and environmental factors likely contribute to this disparity, but biological influences may play a discerning role.

Evidence exists for genomic features contributing to asthma pathogenesis, which in part relates to innate immunological characteristics such as variation in host defense genes [5]. As conveyed in Peden DB 2002, with rapid urbanization over the last few decades, some scientists believe that genes previously protecting humans from parasitic infection may now contribute to a 'misdirected' response to environmental agents [6]. Individuals residing in lower socioeconomic urban environments may experience a higher burden of environmental risk factors for allergy or asthma. Thus, evaluating genetic susceptibility to asthma/allergy in such vulnerable populations can help to direct public health prevention and treatment efforts.

A variety of review papers describe genes associated with allergy/asthma [6-10]. Ober et al. [7] list genes associated with asthma or atopy in more than 10 studies. This study evaluated 8 of these genes (*IL4, IL13, TNF-α, HLA-DRB1, HLA-DQB1, FCER1B/MS4A2, CD14, ADAM33*) as well as 3 glutathione-s-transferase genes (*GSTM1, GSTP1*, and *GSTT1*) for association with asthma/allergy among urban-residing African Americans. We note some key aspects of the biological significance of the genes evaluated.

Many of these genes play roles in the Th2 response to antigens, airway reactivity, or non-specific modulation of inflammation [6]. The Th2 response is more characteristic of asthma in children compared to adults [6,11] and typically accompanied by elevated immunoglobulin E (IgE) response to allergen [12]. Genes within the chromosome 5q cytokine cluster have demonstrated strong associations with asthma/allergy, most notably for *IL4 *and *IL13 *[2,7,13-17]. Associations with total serum IgE have also been observed, but with variable findings across ethnicity [18]. Also located on chromosome 5, the C-159T single nucleotide polymorphism (SNP) within the cluster of differentiation (*CD14*) gene has been associated with asthma, asthma severity [16,19], and total serum IgE [20,21]. The CD14 cell surface receptor mediates host interactions with endotoxin [11], suggesting a role in response to environmental stress. Another gene encoding a proinflammatory cytokine is tumor necrosis factor alpha (*TNF-α*), which has been associated with eosinophilic inflammation during lower respiratory tract infection with respiratory syncytial virus (RSV) in children [22].

Relevant to airway reactivity, a disintegrin and metalloprotease 33 (*ADAM33*) is expressed in bronchial tissue and whole lung [23,24]. *ADAM33 *SNPs have been associated with airway remodeling and bronchial hyper-responsiveness [25], but have varying associations with asthma/allergy and total serum IgE across ethnic populations [16,23,26].

Human Leukocyte Antigen (*HLA*) class II genes relate to non-specific modulation of inflammation. *HLA-DRB1 *and *HLA-DQB1 *SNPs and haplotypes have been associated with a higher risk of toluene diisocyanate-induced occupational asthma [27], total serum IgE in Iranian subjects [28], atopy in Northern Chinese [29], Dermatophagoides Spp.-sensitive asthma in Venuezuelan individuals [30], and asthma severity in Whites in the United States [31], suggesting a broad role for these genes in asthma pathogenesis across ethnic groups.

The high affinity multimeric surface receptor for IgE (Fcepsilon R1beta) is essential for IgE-mediated acute allergic response, and is thereby relevant for asthma pathogenesis [32]. Altered transcription of FcepsilonRlbeta correlates to the MS4A2 membrane-spanning 4-domains, subfamily A, member 2 (*MS4A2*), also known as "Fc fragment of IgE, high affinity I, receptor for beta polypeptide (*FCER1B*)". *MS4A2 *SNPs and haplotypes have been associated with asthma in various populations [33-35].

Outside of the host immune response framework are the glutathione S-transferase (GST) genes, a superfamily of genes that catalyze the conjugation of reduced glutathione to electrophilic and hydrophobic compounds [36]. The conjugation of glutathione is important for the metabolism or detoxification of therapeutic drugs, environmental toxins, and products of oxidative stress, relevant to asthma [37,38]. A recent meta-analysis of GST genes and asthma phenotypes reported that *GSTM1 *and *GSTT1 *showed increased asthma risk associated with the null genotype but that heterogeneity across studies and publication bias was a major limitation [39]. In this paper, we consider key asthma candidate genes for association with asthma and allergy phenotypes among African American children living in Detroit, Michigan.

## Methods

### Study population and measurements

This study involved participants of the Mechanistic Indicators of Asthma (MICA) study, conducted by the United States Environmental Protection Agency between November 2006 and January 2007 in Detroit, Michigan, as previously described [40,41]. This was a cross-sectional study based on a stratified sample of children using two strata: children with asthma and children without asthma, selected in an approximately 1:1 ratio. A total of 205 children age 9-13 years old participated in the clinical study. In order to reduce the genetic heterogeneity of subjects in our analysis, while maximizing the total number of subjects studied, only African American children (N = 141) were selected for analysis. The study design and protocols were approved by the Institutional Review Boards at Henry Ford Health System (Detroit, MI), Westat Inc. (Rockville, MD), and the University of North Carolina at Chapel Hill (Chapel Hill, NC - US EPA's IRB of record). Written consent was obtained from guardians, and written assent was obtained from each child, with an oral review of both consent and assent prior to study enrollment.

For the analyses presented in this paper, hospital records were evaluated to determine doctor diagnosis of asthma for each study participant. To verify genetic associations with doctor diagnosis, two additional variables were created based on parental questionnaire data: current asthma (yes/no), and medication use (yes/no). Current asthma was defined based on the question "Have you had an asthma attack within the last 12 months?" Medication use was defined based on subject reports of asthma medication use.

Allergy-related traits were considered as separate phenotypes. This included total serum IgE, allergen-specific IgE (Phadiatop), testing positive for food allergens based on IgE levels, and absolute eosinophils. Total IgE was measured in serum samples using the ImmunoCAP 250 autoanalyzer (Phadia, Portage, MI, USA). Allergen specific IgE was measured in serum using a multi-allergen screen, which detects the presence (positive/negative) and relative level of antibody specific IgE for a panel of at least 15 common aeroallergens expressed as the Phadiatop level. The food allergen screening test included the 6 most common allergy-provoking foods according to the Food and Drug Administration: cows's milk protein, egg white, wheat, codfish, peanut, and soybean. Eosinophil antibodies were measured in blood samples collected from nonfasting children using standard methods as mandated by Clinical Laboratory Improvement Amendments (CLIA).

### Genotyping

A total of 53 SNPs were selected for genotyping. The candidate SNPs for this study were selected based upon: 1) a literature search for gene regions having multiple, independently-replicated associations with asthma, 2) published variability within African ancestry populations, 3) minimal evidence of LD within African ancestry populations, and 4) methodological considerations. For the first criterion, the primary source for the original SNP search was Ober et al., 2006 [7]. For the second criterion, the SNPs were selected by querying NCBI databases for polymorphisms with minor allele frequency > 10% in African ancestry samples. This was done because approximately 85% of the individuals in the MICA study are African American, only African Americans were analyzed in this report, and as noted in Chang et al. 2009 [42], African ancestry groups have substantially different allele frequencies compared to Whites for some variants. For the third criterion, although we aimed to select SNPs with as little LD as possible, this could not be obtained for all of the genes evaluated. For the fourth criterion, implemented due to methodological considerations of the available genotyping technology, we preferentially chose SNPs within regions of low surrounding SNP density. This fourth criterion was necessary for the HLA gene regions.

Total genomic DNA was isolated from white blood cell pellets using the QiagenFlexiGene DNA kit (Qiagen, Valencia, CA). Additionally, blood clot samples were homogenized using a PowerGen 125 homogenizer (Fisher Scientific, Waltham, MA) and total gDNA was isolated from the homogenate using the QiagenFlexiGene DNA kit. Quantitation of gDNA for each sample was performed using the Nanodrop-1000 (Nanodrop Technologies, Wilmington, DE). Pre-Designed TaqManSNP Genotyping Assay, Custom TaqMan SNP Genotyping Assay or TaqMan Drug Metabolism Genotyping Assay (Applied Biosystems, Inc., Foster City, CA) was selected and designed for each SNP of interest using manufacturer's specificity protocol. Custom TaqMan SNP sequences were submitted via FileBuilder software (Applied Biosystems, Inc.) for assay design. Pre-Designed and Custom TaqMan SNP Genotyping Assays were run with TaqMan Genotyping Master Mix (Applied Biosystems, Inc.) according to the manufacturer suggested cycling conditions. The TaqMan Drug Metabolism Genotyping Assays were run with TaqMan Universal PCR Master Mix (Applied Biosystems, Inc.), also according to the manufacturer suggested cycling conditions. SNP genotypes were determined using 1.0 - 5.0 ng of gDNA template and suggested master mix on the ABI PRISM 7900 Sequence Detection System (Applied Biosystems, Inc.). All SNP PCR runs contained an internal no-template control in order to determine the absence of cross contamination. SNP genotyping was performed in duplicate for ten percent of the DNA samples as a quality control measure to ensure identical genotypes between identical samples. Mismatched genotypes were run a third time to resolve discrepancies.

SNPs missing more than 3% of genotype results and individuals missing more than 10% of their data were excluded from further analyses. Remaining SNPs were tested for consistency with Hardy-Weinberg Equilibrium (HWE) among non-asthmatics using a threshold *p*-value of 0.001. Statistical quality control was completed using *PLINK *version 1.06 [43,44].

### Statistical analysis

Continuous outcomes (total serum IgE, Phadiatop, food allergen-specific IgE, and eosinophils) were log-transformed and assessed for normal distribution using the Shapiro-Wilk test. Log-transformed Phadiatop, food allergen-specific IgE, and eosinophils did not approximate normal distribution and were evaluated as the dichotomous terms based on clinical cut-points used in other studies. Specifically, subjects with Phadiatop values ≥ 0.35 kIU/L were designated as atopic [41]; subjects with food allergen-specific IgE ≥ 0.35 kIU/L were classified as having tested positive for a panel of food allergens [45]; and subjects with absolute eosinophils ≥ 0.40 K/uL were categorized as having eosinophilia.

The association between each variant and asthma/allergy was evaluated through linear regression for continuous dependent variables (total serum IgE) or logistic regression for dichotomous dependent variables (asthma, atopy, testing positive for food allergens, and eosinophilia), following an additive genetic model. False discovery rate (FDR) was employed to correct for multiple testing [46]. A Q value ≤ 0.25 was used to indicate the minimum FDR where the association was considered significant. Analyses were completed using *PLINK *[43,44] and *R *version 2.12.0 [47]. Linkage disequilibrium (LD) across SNPs was examined for each chromosome or gene in *Haploview *version 4.1 [48]. For perspective, genotyped SNPs were evaluated for LD with HapMap SNPs from the individuals of African ancestry from the South West United States (ASW) population.

Significant SNP associations were followed up with haplotype analyses. SNPs were phased in *PLINK *[43,44] using the expectation-maximization (E-M) algorithm to assign individual haplotypes. Haplotypes were then evaluated for association with asthma/allergy through linear and logistic regression. To evaluate the consistency of haplotype estimation and association across software, *PHASE *version 2.1 [49,50] and *Haplostats *version 1.4.4 [51] were employed to estimate haplotype assignments per individual and haplotype associations with asthma related traits, respectively. *Haplostats *haplo.scan [52] was implemented to identify the locus or loci within each haplotype demonstrating the strongest association with a particular trait. All haplotype analyses were run if the genes had one or more statistically significant SNP associations with the phenotypes of interest, after correction for multiple testing. FDR was also applied to the haplotype analyses. Age, sex, body mass index, and urinary cotinine levels were evaluated for effect measure modification using the Breslow-Day test of homogeneity of effect estimates across covariate categories. Confounding was subsequently evaluated using the percent change in effect estimate criterion [45,53].

In addition to the haplotype analyses, SNP association analyses were complimented by constructing a genetic risk score (GRS), using a linear weighting of 0, 1, or 2 for genotypes containing 0, 1, or 2 risk alleles, respectively. For this model only, risk alleles were considered those that increased the risk of asthma in the independent SNP models. For example, because rs1871042 T corresponded to a lower risk of asthma, rs1871042 C was considered the risk allele for the GRS. The purpose of the GRS was to assess the cumulative effect of the key SNPs in our study on the odds of asthma in this population. A GRS has been used in studies of heart disease and diabetes using a variety of methods [54-57]. We followed similar methodology to that of a study describing a GRS to predict type 2 diabetes [58]. The program *Tagger *[59] was employed to select a minimal set of SNPs from each gene. Selected SNPs were considered independent and equally weighted. The GRS was tested for normal distribution using the Shapiro-Wilk normality test and a trend test was applied to evaluate the linearity of the relationship between the number of risk alleles and the odds of asthma.

## Results

### Quality control

Among the 10% of samples run in duplicate, 100% obtained identical results. A total of 41 of the 53 SNPs (77%) passed statistical quality control. Association with asthma/allergy was evaluated for the following genes (SNPs): *GSTM1 *(rs17672 C/T, rs412543 G/C, rs3815029 G/C), *IL13 *(rs1800925 C/T, rs1295686 A/G, rs20541 C/T, rs848 G/T, rs2069750 G/T), *IL4 *(rs2070874 C/T, rs734244 G/A, rs2243267 G/C, rs2243270 G/A, rs2243290 C/A), *CD14 *(rs2563298 C/A, rs5744456 T/A, rs2569190 G/A, rs2569191 T/C, rs3138078 G/T), *TNF *(rs1800629 G/A, rs3093662 A/G, rs3093664 A/G, rs3093665 A/C, rs3093668 G/C), *HLA-DRB1 *(rs9269701 G/A, rs9269743 A/G, rs9269841 G/A), *HLA-DQB1 *(rs4993986 C/G), *MS4A2 *(rs574700 C/T, rs1441586 T/C, rs556917 A/T, rs502581 C/A, rs502419 G/A), *GSTP1 *(rs6591256 A/G, rs17593068 G/T, rs1695 A/G, rs1871042 C/T, rs947895 C/A), *ADAM33 *(rs2787094 G/C, rs543749 G/T, rs44707 A/C, rs2271511 C/T), and *GSTT1 *(null vs. present genotype).

### Demographics and phenotypic/genotypic differences by ethnic group

Among the 141 individuals analyzed, 63 (44.7%) were female and 51 (36.2%) had a body mass index of 25 or higher. A total of 64 (49.6%) individuals were asthmatic, 92 (66%) atopic, 54 (39.7%) tested positive for a panel of food allergens, and 24 (17%) were designated as having eosinophilia. Of the 53 SNPs subjected to quality control, 12 SNPs were excluded due to missing > 3% of genotyping data (N = 7) or failed HWE (N = 5). Information on the remaining 41 SNPs and *GSTT1 *-null variant was available for 141 African American subjects. The mean total serum IgE was 4.63 kU/L (standard deviation = 1.62 kU/L). Total serum IgE, atopy, testing positive for food allergens, and eosinophilia were significantly associated with asthma (Table 1) and evaluated as outcomes in separate statistical models. No effect measure modification by covariates was observed. Sex was found to be the only significant confounder in the SNP and haplotypes models and was included as a covariate.

**Table 1:** Association between asthma and related traits

Trait	OR (95% CI)	*p*
Total serum IgE	1.60 (1.24, 2.07)	< 0.0001
Atopy	3.09 (1.39, 6.87)	0.006
Food allergen test positive	2.04 (0.99, 4.20)	0.053
Eosinophilia	3.56 (1.30, 9.73)	0.013

† Logistic regression employed to estimate the Odds Ratio (OR) and 95% Confidence Interval (CI) of the Odds Ratio; *p: p-value*.

### SNP associations

**Figure 1:**
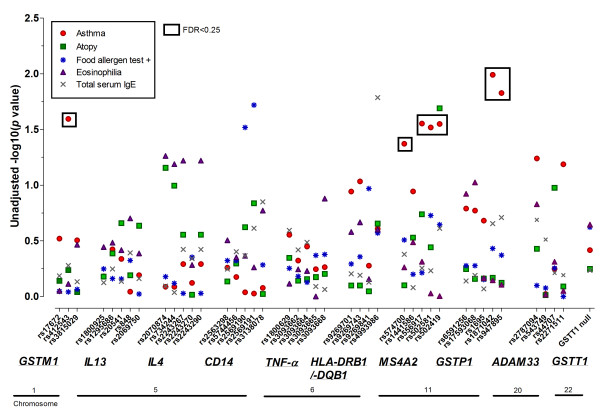
**Segmented Manhattan plot displaying SNP associations with asthma/allergy traits**. Negative log-transformed *p*-value (-log(*p*)) indicative of statistical significance for the association between each SNP and trait, where higher -log(*p*) values correspond to greater statistical significance. SNPs significant based on FDR < 0.25 indicated with a black box.

**Figure 2:**
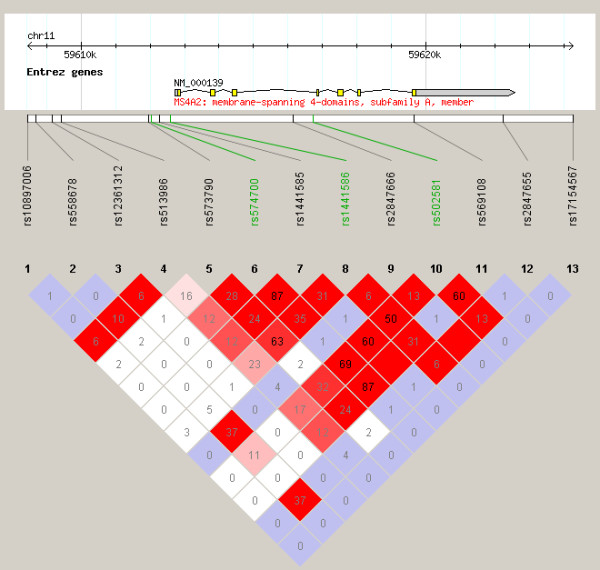
**Linkage disequilibrium (LD) observed across SNPs of *MS4A2*. r^2 ^values displayed in triangle plot represent strength of LD between SNPs**. Color scheme: white (weakest LD): D' < 1, LOD < 2; blue: D' = 1, LOD < 2; shades of pink/red: D' < 1, LOD≠2; bright red (strongest LD): D' = 1, LOD≠2. SNPs in study population indicated in green, HapMap SNPs in black. HapMap data specific to the individuals of African ancestry from the South West United States (ASW) population.

**Figure 3:**
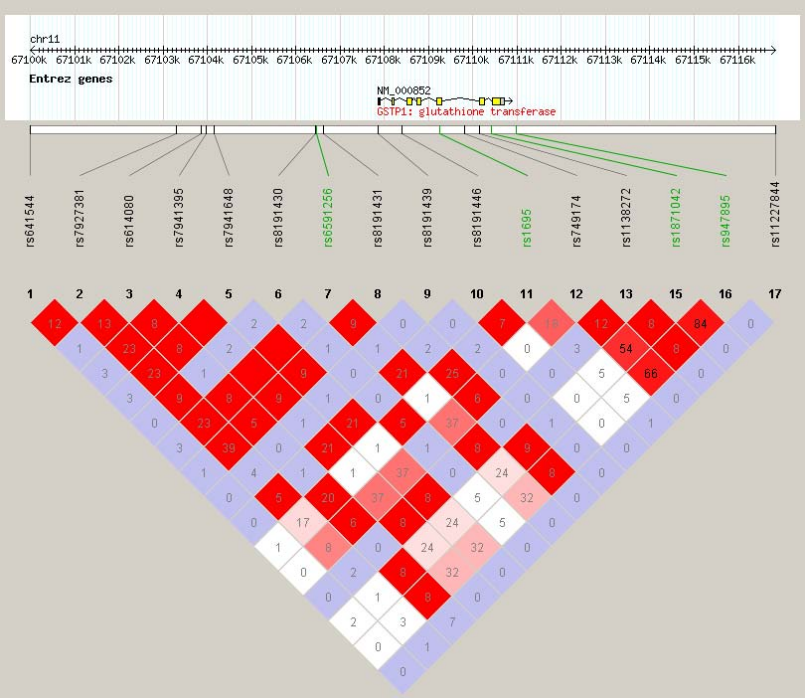
**Linkage disequilibrium (LD) observed across SNPs of *GSTP1***. r^2 ^values displayed in triangle plot represent strength of LD between SNPs. Color scheme: white (weakest LD): D' < 1, LOD < 2; blue: D' = 1, LOD < 2; shades of pink/red: D' < 1, LOD≠2; bright red (strongest LD): D' = 1, LOD≠2. SNPs in study population indicated in green, HapMap SNPs in black. HapMap data specific to the individuals of African ancestry from the South West United States (ASW) population.

The number of subjects analyzed for each phenotype was 129, 138, 136, 140, and 140, for asthma, atopy, testing positive for food allergy, eosinophilia, and total serum IgE phenotypes, respectively. Statistically significant associations were only observed for the asthma phenotype, for SNPs in *GSTM1, MS4A2*, and *GSTP1*, after correction for multiple testing (Figure 1, Table 2). *GSTM1 *rs412543 C carriers were almost 3 times as likely to have asthma compared to individuals without the C allele. All of the *MS4A2 *SNPs evaluated were associated with a lower odds of asthma, and the associations were statistically significant for rs556917 A/T, rs502581 C/A, and rs502419 G/A. Strong linkage disequilibrium existed across *MS4A2*, in particular for rs556917, rs502581, and rs502419 (r^2 ^> 0.7, Figure 2). The tightly linked *GSTP1 *SNPs rs1871042 C/T and rs947865 C/A (Figure 3) were associated with a lower odds of asthma.

**Table 2:** Association between SNPs and asthma

Gene	Chr	Position	SNP	MAF	Odds Ratio (95% CI)	*Unadjusted p*	*FDR*
*GSTM1*	1	108482748	rs17672 C/T	0.16	1.47 (0.71, 3.04)	0.302	0.673
	1	110031467	rs412543 G/C	0.12	2.89 (1.14, 7.32)	0.025	**0.207**
	1	110031479	rs3815029 G/C	0.17	0.69 (0.33, 1.42)	0.312	0.673

*MS4A2*	11	59612059	rs574700 C/T	0.18	0.50 (0.23, 1.07)	0.042	0.249
	11	59612604	rs1441586 T/C	0.43	0.56 (0.25, 1.25)	0.114	0.390
	11	59615288	rs556917 A/T	0.34	0.47 (0.22, 0.97)	0.028	**0.207**
	11	59616754	rs502581 C/A	0.26	0.50 (0.24, 1.02)	0.030	**0.207**
	11	59622751	rs502419 G/A	0.24	0.45 (0.21, 0.93)	0.028	**0.207**

*GSTP1*	11	67106475	rs6591256 A/G	0.43	0.66 (0.31, 1.38)	0.162	0.495
	11	67107508	rs17593068 G/T	0.43	0.71 (0.34, 1.50)	0.169	0.495
	11	67109265	rs1695 A/G	0.44	1.61 (0.75, 3.42)	0.208	0.569
	11	67110420	rs1871042 C/T	0.30	0.44 (0.22, 0.91)	0.010	**0.207**
	11	67110982	rs947895 C/A	0.29	0.44 (0.22, 0.91)	0.015	**0.207**

† 41 SNPs evaluated as well as the *GSTT1 *deletion. Logistic regression adjusted for sex and performed in *PLINK *version 1.06. SNP major allele/minor allele noted, in that order. MAF: Minor allele frequency; Chr: Chromosome, 95% CI: 95% Confidence Intervals of the odds ratio, *p: p-value*. Statistical significance after correction for multiple comparisons evaluated using False Discovery Rate (FDR). FDR ≤ 0.25 denoted statistically significant associations, marked in bold. Data presented for genes with one or more statistically significant associations. Models included 129 individuals.

### Haplotype associations

The statistically significant SNP associations were complimented by gene-specific haplotype analyses, presented in Table 3. *GSTM1 *haplotypes CCG and CGC corresponding to SNPs rs17672, rs412543, and rs3815029, showed contrasting direction of associations with asthma. Specifically, the CCG haplotype was associated with a 3 fold increase in the odds of asthma whereas the CGC haplotype corresponded to an Odds Ratio (OR) of 0.36. The frequency of both of these haplotypes was approximately 10%. The *MS4A2 *TCTAA haplotype offered a protective association with asthma compared to the CTACG (Table 3). Sliding window analyses in *Haplostats *indicated that 3 tightly linked SNPs (*MS4A2*-ACG haplotype for rs556917, rs502581, and rs502419) drove the association with asthma (simulated *p *values = 0.03, 0.033, 0.026 for rs556917, rs502581, and rs502419, respectively, 1000 simulations). *GSTP1 *AGATA (OR = 0.28, *p *= 0.027) and *GSTP1 *AGGCC (OR = 2.23, *p *= 0.018) displayed contrasting associations with asthma. Sliding window analyses in *Haplostats *identified loci rs1695, rs1871042, and rs947895 to be the strongest SNPs driving the associations, based on the maximum scan statistics. However, the locus-scan statistic *p *values and max-stat simulated global *p *value were not statistically significant (*p *> 0.05 for 1000 simulations).

**Table 3:** Association between haplotypes and asthma

Gene	Chr	BP1	BP2	SNP1	SNP2	Haplotype	Frequency	Odds Ratio	*Unadjusted p*	*FDR*
*GSTM1*	1	108482748	110031479	rs17672	rs3815029	CCG	0.10	3.01	0.05	**0.210**
	1	108482748	110031479	rs17672	rs3815029	CGC	0.11	0.36	0.04	**0.209**
	1	108482748	110031479	rs17672	rs3815029	CGG	0.64	0.82	0.506	0.781
	1	108482748	110031479	rs17672	rs3815029	TCC	0.01	4.48	0.298	0.626
	1	108482748	110031479	rs17672	rs3815029	TGC	0.05	1.58	0.508	0.781
	1	108482748	110031479	rs17672	rs3815029	TGG	0.09	1.18	0.759	0.839

	11	59612059	59622751	rs574700	rs502419	CCACG	0.08	1.63	0.286	0.626
	11	59612059	59622751	rs574700	rs502419	CCTAA	0.07	0.86	0.758	0.839
	11	59612059	59622751	rs574700	rs502419	CCTCA	0.01	1.02	0.982	0.982
*MS4A2*	11	59612059	59622751	rs574700	rs502419	CCTCG	0.07	0.78	0.595	0.781
	11	59612059	59622751	rs574700	rs502419	CTACG	0.57	1.58	0.114	0.399
	11	59612059	59622751	rs574700	rs502419	TCTAA	0.14	0.43	0.032	**0.209**
	11	59612059	59622751	rs574700	rs502419	TCTAG	0.04	1.04	0.96	0.982

	11	67106475	67110982	rs6591256	rs947895	AGACC	0.27	1.2	0.519	0.781
	11	67106475	67110982	rs6591256	rs947895	AGATA	0.07	0.28	0.027	**0.209**
	11	67106475	67110982	rs6591256	rs947895	AGATC	0.01	0.56	0.642	0.793
*GSTP1*	11	67106475	67110982	rs6591256	rs947895	AGGCC	0.21	2.23	0.018	**0.209**
	11	67106475	67110982	rs6591256	rs947895	GTACC	0.18	0.82	0.573	0.781
	11	67106475	67110982	rs6591256	rs947895	GTATA	0.02	0.19	0.201	0.603
	11	67106475	67110982	rs6591256	rs947895	GTGCC	0.03	1.59	0.562	0.781
	11	67106475	67110982	rs6591256	rs947895	GTGTA	0.19	0.70	0.288	0.626

† Logistic regression adjusted for sex and performed in PLINK version 1.06. SNP1: SNP of left-most (5') SNP in haplotype, SNP2: SNP of right most (3') SNP in haplotype. ß: Beta estimate, *p: p-*value. Statistical significance after correction for multiple comparisons evaluated using False Discovery Rate (FDR). FDR ≤ 0.25 denoted statistically significant associations, marked in bold. Models included 129 individuals.

### Chromosome-wide haplotype association

Chromosome 11 haplotypes displayed the strongest statistically significant associations with asthma and were subsequently evaluated for a chromosome-wide haplotype effect on asthma. The chromosome 11 haplotype CTACGAGGCC (corresponding to *MS4A2 *rs574700, rs1441586, rs556917, rs502581, rs502419 and *GSTP1 *rs6591256, rs17593068, rs1695, rs1871042, rs947895) was associated with a nearly five-fold increase in the odds of asthma (OR = 4.8, *p *= 0.007). The frequency of this haplotype was 10% in our population. At the time this work was conducted, *PLINK *software did not include computation of 95% confidence intervals for haplotype associations.

### Genetic risk score

SNPs significantly associated with elevated odds of asthma were evaluated in concert by constructing a genetic risk score. This score combined data for the following SNPs: *GSTM1 *rs412543, *MS4A2 *rs556917 and rs502419 (tagging rs502581), and *GSTP1 *rs947895 (tagging rs1871042). The *MS4A2 *SNPs rs556917 and rs502419 did not tag each other, and were both included in the GRS. This was consistent with modified settings of the *Tagger *program (data not shown).

The genetic risk score ranged from 0 to 7 variant alleles carried by an individual and approximated normal distribution (W = 0.98, Z = 1.142, *p *= 0.126). The GRS was statistically significantly associated with a higher odds of asthma (OR = 1.61, 95% Confidence Interval (CI) = 1.21, 2.13; *p *= 0.001). Incremental increases in the GRS corresponded to higher total serum IgE and higher odds of atopy and eosinophilia, but these results were not statistically significant.

## Discussion and Conclusion

### Genetic associations with asthma

In this study, we evaluated previously identified variants for association with asthma/allergy among African American children living in Detroit, Michigan. Of the 41 SNPs evaluated in statistical analyses, SNPs within *GSTM1, MS4A2*, and *GSTP1 *were associated with asthma. Chromosome 11 SNPs, in particular the *MS4A2 *-CTACG/*GSTP1 *-AGGCC haplotype, played the greatest role in genetic predisposition to asthma in this population. When risk alleles were evaluated in concert using a genetic risk score, SNPs predisposing individuals to asthma exhibited an expected cumulative effect. The genetic risk score (or genetic risk index) has been employed to understand individual susceptibility to obesity [60], cardiovascular disease [61-63], coronary artery disease [64], diabetes [58], serum uric acid concentration [65], multiple sclerosis [66], and rheumatoid arthritis [67]. Some studies have evaluated a weighted GRS, where each SNP included in the score is weighted by a beta coefficient from a meta-analysis of SNPs associated with disease, as was done in Cornelis et al., 2009 [58]. To our knowledge, a meta-analysis providing sufficient data for the construction of a GRS for asthma/allergy was not available at the time these analyses were completed.

### Functional significance and role in asthma etiology

The observation that variation in *MS4A2 *was significantly associated with a higher risk of asthma is consistent with *MS4A2*'s involvement in allergic disease [32]. SNPs within *MS4A2 *have previously been associated with atopy via regulation of Fc epsilon RI expression [68]. A recent study among Australians, Dutch, and Danish individuals found weak evidence for rs502581 to be associated with IgE [69]. We did not identify significant associations between this SNP and IgE in our study population, but we note the direction of effect was consistent with what has been reported, the A allele corresponding to lower IgE levels (β = -0.14, 95% CI = -0.64, 0.36, *p *= 0.587) and the T allele corresponding to higher IgE levels. In our study population, the A allele was the risk allele tested due to default parameters in *PLINK*.

*GSTP1's *role in xenobiotic metabolism and antioxidation is also consistent with asthma etiology, although it is likely to play a different role in asthma pathogenesis compared to *MS4A2*. Variation in this gene may result in differing metabolism of environmental toxins across individuals. We speculate that individuals with multiple risk alleles across *MS4A2 *and *GSTP1 *genes are more susceptible to harmful effects of environmental toxins, and that this sensitivity may contribute to the development of asthma or asthma exacerbation. Additional investigations incorporating environmental data are required to confirm this hypothesis. We did not observe significant associations between the functional sequence variant in *GSTP1 *at codon 15 (Ile105Va - rs1695). The role of this variant in asthma/allergy has been inconsistently reported in the literature, possibly due to heterogeneity of environmental exposures [70]. Other studies have reported that this variant is tagged by rs947895 in White populations [71], which corresponded to a significant association with asthma in our study. However, the genetic variation that is tagged by this SNP in our African ancestry population may be less than what has been reported in other ethnic groups.

### Supporting analyses

Asthma is a complex trait that is variably defined across studies. Thus, we compared our definition of asthma with slightly variable phenotypes, current asthma (yes/no) and asthma medication use (yes/no) based on parental questionnaire data. The association between SNPs, haplotypes, or the GRS and asthma was consistent when evaluated for current asthma or medication use, with regards to the direction of association (odds ratio estimates) and statistical significance (*p*-values).

### Limitations

Our study population was largely atopic (67%) and over 39% tested positive for response to a panel of food allergens. These statistics limit the generalizability of our findings to children of similar age in the United States. However, the associations discussed here may be informative for other vulnerable populations or high risk groups where the prevalence of asthma is high.

The sample size of 141 limited our power to detect statistically significant associations after correction for multiple testing. Because of the small number of SNPs genotyped, we were unable to capture much of the genomic landscape relevant to the phenotypes of interest. Thus, unmeasured genetic risk factors that play a role in asthma/allergy susceptibility were inadequately captured in this study. The small number of SNPs also limited our ability to comprehensively assess population stratification through principal components analysis using genome wide data or ancestry informative markers (AIMS). We relied on self-reported ethnicity to reduce issues of genetic heterogeneity. For these reasons, caution is warranted when assessing the generalizability of our study findings to other independent cohorts.

The candidate genes were selected for genotyping before the publication of the *DENND1B *association with asthma in a genome wide association study of African-ancestry populations [72]. *DENND1B *is expressed by natural killer cells and dendritic cells, encoding a protein that interacts with the TNF-α receptor. Although we do not present data on this gene, we note that significant associations between the measured *TNF-α *SNPs and asthma were not observed in this study population.

### Future directions

This report did not incorporate environmental risk factors for asthma. It is likely that environmental agents interact with a person's genetic predisposition or provide an additional burden on the genetically susceptible group, increasing the odds of asthma/allergy or exacerbating symptoms. Future work should incorporate a greater number of subjects and SNPs, and evaluate SNPs and environmental exposures in concert as they relate to asthma, asthma severity and asthma exacerbation. It remains particularly important to test and validate associations in populations with a high disease burden.

## Competing interests

The authors declare that they have no competing interests.

## Authors' contributions

BRJ wrote and revised the paper and performed the statistical analyses with input from DR, PE, SE, ECH, JEG. DR contributed to the SNP selection and quality assessment for SNP analysis. KAL supervised and contributed to genotyping data at the Integrated Laboratory Systems, RTP, NC. EH was involved in the collection of questionnaire and immunological data. JEG is the principal investigator of the MICA study. ECH provided supervision of the research and revision of the manuscript. All authors reviewed and approved the final manuscript.

## Pre-publication history

The pre-publication history for this paper can be accessed here:

http://www.biomedcentral.com/1471-2350/12/25/prepub

## References

[B1] Busse WW Lemanske RF Jr Asthma N Engl J Med 2001 344 5 350 362 10.1056/NEJM200102013440507 11172168

[B2] Barnes KC Grant AV Hansel NN Gao P Dunston GM African Americans with asthma: genetic insights Proc Am Thorac Soc 2007 4 1 58 68 10.1513/pats.200607-146JG 17202293 PMC2647616

[B3] Scirica CV Celedon JC Genetics of asthma: potential implications for reducing asthma disparities Chest 2007 132 5 Suppl 770S 781S 10.1378/chest.07-1905 17998341

[B4] Joseph CL Williams LK Ownby DR Saltzgaber J Johnson CC Applying epidemiologic concepts of primary, secondary, and tertiary prevention to the elimination of racial disparities in asthma J Allergy Clin Immunol 2006 117 2 233 240 quiz 241-232 10.1016/j.jaci.2005.11.004 16461121 PMC1904504

[B5] Gao L Tsai YJ Grigoryev DN Barnes KC Host defense genes in asthma and sepsis and the role of the environment Curr Opin Allergy Clin Immunol 2007 7 6 459 467 10.1097/ACI.0b013e3282f1fb9a 17989521

[B6] Peden DB Influences on the development of allergy and asthma Toxicology 2002 181-182 323 328 10.1016/S0300-483X(02)00301-3 12505333

[B7] Ober C Hoffjan S Asthma genetics 2006: the long and winding road to gene discovery Genes Immun 2006 7 2 95 100 10.1038/sj.gene.6364284 16395390

[B8] Holloway JW Yang IA Holgate ST Genetics of allergic disease J Allergy Clin Immunol 2010 125 2 Suppl 2 S81 94 10.1016/j.jaci.2009.10.071 20176270

[B9] Vercelli D Advances in asthma and allergy genetics in 2007 J Allergy Clin Immunol 2008 122 2 267 271 10.1016/j.jaci.2008.06.008 18619666

[B10] Vercelli D Discovering susceptibility genes for asthma and allergy Nat Rev Immunol 2008 8 3 169 182 10.1038/nri2257 18301422

[B11] Kleeberger SR Peden D Gene-environment interactions in asthma and other respiratory diseases Annu Rev Med 2005 56 383 400 10.1146/annurev.med.56.062904.144908 15660518

[B12] Platts-Mills TA Allergens and asthma Allergy Proc 1990 11 6 269 271 10.2500/108854190778879729 2292373

[B13] Rosenwasser LJ Klemm DJ Dresback JK Inamura H Mascali JJ Klinnert M Borish L Promoter polymorphisms in the chromosome 5 gene cluster in asthma and atopy Clin Exp Allergy 1995 25 Suppl 2 74 78 discussion 95-76 10.1111/j.1365-2222.1995.tb00428.x 8590350

[B14] Walley AJ Cookson WO Investigation of an interleukin-4 promoter polymorphism for associations with asthma and atopy J Med Genet 1996 33 8 689 692 10.1136/jmg.33.8.689 8863163 PMC1050705

[B15] Pinto LA Stein RT Ribeiro JD Genetic associations with asthma and virus-induced wheezing: a systematic review J Bras Pneumol 2009 35 12 1220 1226 10.1590/S1806-37132009001200010 20126925

[B16] de Faria IC de Faria EJ Toro AA Ribeiro JD Bertuzzo CS Association of TGF-beta1, CD14, IL-4, IL-4R and ADAM33 gene polymorphisms with asthma severity in children and adolescents J Pediatr (Rio J) 2008 84 3 203 210 10.2223/JPED.1783 18425216

[B17] Haller G Torgerson DG Ober C Thompson EE Sequencing the IL4 locus in African Americans implicates rare noncoding variants in asthma susceptibility J Allergy Clin Immunol 2009 124 6 1204 1209 e1209 10.1016/j.jaci.2009.09.013 19910025 PMC3984460

[B18] Basehore MJ Howard TD Lange LA Moore WC Hawkins GA Marshik PL Harkins MS Meyers DA Bleecker ER A comprehensive evaluation of IL4 variants in ethnically diverse populations: association of total serum IgE levels and asthma in white subjects J Allergy Clin Immunol 2004 114 1 80 87 10.1016/j.jaci.2004.05.035 15241348

[B19] Walley AJ Wiltshire S Ellis CM Cookson WO Linkage and allelic association of chromosome 5 cytokine cluster genetic markers with atopy and asthma associated traits Genomics 2001 72 1 15 20 10.1006/geno.2000.6435 11247662

[B20] Baldini M Lohman IC Halonen M Erickson RP Holt PG Martinez FD A Polymorphism* in the 5' flanking region of the CD14 gene is associated with circulating soluble CD14 levels and with total serum immunoglobulin E Am J Respir Cell Mol Biol 1999 20 5 976 983 10226067 10.1165/ajrcmb.20.5.3494

[B21] Vercelli D Baldini M Stern D Lohman IC Halonen M Martinez F CD14: a bridge between innate immunity and adaptive IgE responses J Endotoxin Res 2001 7 1 45 48 11521081

[B22] Choi J Callaway Z Kim HB Fujisawa T Kim CK The role of TNF-alpha in eosinophilic inflammation associated with RSV bronchiolitis Pediatr Allergy Immunol 2010 10.1111/j.1399-3038.2009.00908.x 20088864

[B23] Van Eerdewegh P Little RD Dupuis J Del Mastro RG Falls K Simon J Torrey D Pandit S McKenny J Braunschweiger K Association of the ADAM33 gene with asthma and bronchial hyperresponsiveness Nature 2002 418 6896 426 430 10.1038/nature00878 12110844

[B24] Yoshinaka T Nishii K Yamada K Sawada H Nishiwaki E Smith K Yoshino K Ishiguro H Higashiyama S Identification and characterization of novel mouse and human ADAM33s with potential metalloprotease activity Gene 2002 282 1-2 227 236 10.1016/S0378-1119(01)00818-6 11814695

[B25] Lee JH Park HS Park SW Jang AS Uh ST Rhim T Park CS Hong SJ Holgate ST Holloway JW ADAM33 polymorphism: association with bronchial hyper-responsiveness in Korean asthmatics Clin Exp Allergy 2004 34 6 860 865 10.1111/j.1365-2222.2004.01977.x 15196271

[B26] Howard TD Postma DS Jongepier H Moore WC Koppelman GH Zheng SL Xu J Bleecker ER Meyers DA Association of a disintegrin and metalloprotease 33 (ADAM33) gene with asthma in ethnically diverse populations J Allergy Clin Immunol 2003 112 4 717 722 10.1016/S0091-6749(03)01939-0 14564349

[B27] Choi JH Lee KW Kim CW Park CS Lee HY Hur GY Kim SH Hong CS Jang AS Park HS The HLA DRB1*1501-DQB1*0602-DPB1*0501 haplotype is a risk factor for toluene diisocyanate-induced occupational asthma Int Arch Allergy Immunol 2009 150 2 156 163 10.1159/000218118 19439981

[B28] Movahedi M Moin M Gharagozlou M Aghamohammadi A Dianat S Moradi B Nicknam MH Nikbin B Amirzargar A Association of HLA class II alleles with childhood asthma and Total IgE levels Iran J Allergy Asthma Immunol 2008 7 4 215 220 19052351

[B29] Gao J Lin Y Qiu C Liu Y Ma Y Association between HLA-DQA1, -DQB1 gene polymorphisms and susceptibility to asthma in northern Chinese subjects Chin Med J (Engl) 2003 116 7 1078 1082 12890388

[B30] Lara-Marquez ML Yunis JJ Layrisse Z Ortega F Carvallo-Gil E Montagnani S Makhatadze NJ Pocino M Granja C Yunis E Immunogenetics of atopic asthma: association of DRB1*1101 DQA1*0501 DQB1*0301 haplotype with Dermatophagoides spp.-sensitive asthma in a sample of the Venezuelan population Clin Exp Allergy 1999 29 1 60 71 10.1046/j.1365-2222.1999.00461.x 10051703

[B31] Juhn YJ Kita H Bagniewski SM Weaver AL Pankratz VS Jacobson RM Poland GA Severity of childhood asthma and human leukocyte antigens type J Asthma 2007 44 3 163 168 10.1080/02770900701209632 17454332

[B32] Kraft S Rana S Jouvin MH Kinet JP The role of the FcepsilonRI beta-chain in allergic diseases Int Arch Allergy Immunol 2004 135 1 62 72 10.1159/000080231 15316148

[B33] Sharma S Ghosh B Promoter polymorphism in the MS4A2 gene and asthma in the Indian population Int Arch Allergy Immunol 2009 149 3 208 218 10.1159/000199716 19218813

[B34] Sharma S Nagarkatti R C BR Niphadkar PV Vijayan V Sharma SK Ghosh B A_16_C haplotype in the FcepsilonRIbeta gene confers a higher risk for atopic asthma in the Indian population Clin Genet 2004 66 5 417 425 10.1111/j.1399-0004.2004.00333.x 15479187

[B35] Potaczek DP Sanak M Szczeklik A Additive association between FCER1A and FCER1B genetic polymorphisms and total serum IgE levels Allergy 2007 62 9 1095 1096 10.1111/j.1398-9995.2007.01446.x 17686114

[B36] Sayers EW Barrett T Benson DA Bryant SH Canese K Chetvernin V Church DM DiCuccio M Edgar R Federhen S Database resources of the National Center for Biotechnology Information Nucleic Acids Res 2009 37 Database D5 15 10.1093/nar/gkn741 PMC2686545 18940862

[B37] Ercan H Birben E Dizdar EA Keskin O Karaaslan C Soyer OU Dut R Sackesen C Besler T Kalayci O Oxidative stress and genetic and epidemiologic determinants of oxidant injury in childhood asthma J Allergy Clin Immunol 2006 118 5 1097 1104 10.1016/j.jaci.2006.08.012 17088135

[B38] Rahman I Biswas SK Kode A Oxidant and antioxidant balance in the airways and airway diseases Eur J Pharmacol 2006 533 1-3 222 239 10.1016/j.ejphar.2005.12.087 16500642

[B39] Minelli C Granell R Newson R Rose-Zerilli MJ Torrent M Ring SM Holloway JW Shaheen SO Henderson JA Glutathione-S-transferase genes and asthma phenotypes: a Human Genome Epidemiology (HuGE) systematic review and meta-analysis including unpublished data Int J Epidemiol 2010 20032267 10.1093/ije/dyp337 PMC2846443

[B40] Williams AH Gallagher JE Hudgens E Johnson MM Mukerjee S Ozkaynak HA Nea LM EPA observational studies of children’s respiratory health in Detroit and Dearborn, Michigan. Proceedings Air & Waste Management Association's 102nd Annual Conference & Exhibition June 16-17, 2009

[B41] Heidenfelder B Johnson M Hudgens E Inmon J Hamilton RG Neas L Gallagher JE Increased plasma reactive oxidant levels and their relationship to blood cells, total IgE, and allergen-specific IgE levels in asthmatic children J Asthma 2010 47 1 106 111 10.3109/02770900903435964 20100030

[B42] Chang MH Lindegren ML Butler MA Chanock SJ Dowling NF Gallagher M Moonesinghe R Moore CA Ned RM Reichler MR Prevalence in the United States of selected candidate gene variants: Third National Health and Nutrition Examination Survey, 1991-1994 Am J Epidemiol 2009 169 1 54 66 10.1093/aje/kwn286 18936436 PMC2638878

[B43] Purcell S Neale B Todd-Brown K Thomas L Ferreira MA Bender D Maller J Sklar P de Bakker PI Daly MJ PLINK: a tool set for whole-genome association and population-based linkage analyses Am J Hum Genet 2007 81 3 559 575 10.1086/519795 17701901 PMC1950838

[B44] Purcell S PLINK version 1.06 2010 http://pngu.mgh.harvard.edu/~purcell/plink/

[B45] Food Allergies: What you Need to Know 2007 US UFDA http://www.fda.gov/downloads/Food/ResourcesForYou/Consumers/UCM079428.pdf

[B46] Benjamini Y Hochberg Y Controlling the False Discovery Rate - a Practical and Powerful Approach to Multiple Testing J Roy Stat Soc B Met 1995 57 1 289 300

[B47] R Development Core Team R: A Language and Environment for Statistical Computing 2010 R Foundation for Statistical Computing http://www.R-project.org/

[B48] Barrett JC Fry B Maller J Daly MJ Haploview: analysis and visualization of LD and haplotype maps Bioinformatics 2005 21 2 263 265 10.1093/bioinformatics/bth457 15297300

[B49] Stephens M Smith NJ Donnelly P A new statistical method for haplotype reconstruction from population data Am J Hum Genet 2001 68 4 978 989 10.1086/319501 11254454 PMC1275651

[B50] Stephens M Donnelly P A comparison of bayesian methods for haplotype reconstruction from population genotype data Am J Hum Genet 2003 73 5 1162 1169 10.1086/379378 14574645 PMC1180495

[B51] Schaid DJ Rowland CM Tines DE Jacobson RM Poland GA Score tests for association between traits and haplotypes when linkage phase is ambiguous Am J Hum Genet 2002 70 2 425 434 10.1086/338688 11791212 PMC384917

[B52] Cheng R Ma JZ Wright FA Lin S Gao X Wang D Elston RC Li MD Nonparametric disequilibrium mapping of functional sites using haplotypes of multiple tightly linked single-nucleotide polymorphism markers Genetics 2003 164 3 1175 1187 12871923 10.1093/genetics/164.3.1175 PMC1462627

[B53] Maldonado G Greenland S Simulation study of confounder-selection strategies Am J Epidemiol 1993 138 11 923 936 8256780 10.1093/oxfordjournals.aje.a116813

[B54] De Miguel-Yanes JM Shrader P Sullivan LM Fox CS Dupuis J Manning AK Florez JC Wilson PWF D'Agostino RB Cupples LA A Type 2 Diabetes (T2D) Genetic Risk Score Using 37 Single Nucleotide Polymorphisms (SNPs) Significantly Reclassifies T2D Risk beyond Clinical T2D Risk Factors Diabetes 2010 59 A334 A334

[B55] Yiannakouris N A Genetic Score for CHD Risk Prediction J Nutrigenet Nutrige 2008 1 6 280 281

[B56] Dandona S Roberts R Creating a genetic risk score for coronary artery disease Current Atherosclerosis Reports 2009 11 3 175 181 10.1007/s11883-009-0028-4 19361348

[B57] Pankow JS Kao WH North KE Peacock JM Folsom AR Boerwinkle E Prediction of type 2 diabetes by a genetic risk score: The ARIC study Diabetes 2008 57 A331 A332

[B58] Cornelis MC Qi L Zhang C Kraft P Manson J Cai T Hunter DJ Hu FB Joint effects of common genetic variants on the risk for type 2 diabetes in U.S. men and women of European ancestry Ann Intern Med 2009 150 8 541 550 19380854 10.7326/0003-4819-150-8-200904210-00008 PMC3825275

[B59] de Bakker PI Burtt NP Graham RR Guiducci C Yelensky R Drake JA Bersaglieri T Penney KL Butler J Young S Transferability of tag SNPs in genetic association studies in multiple populations Nat Genet 2006 38 11 1298 1303 10.1038/ng1899 17057720

[B60] He M Cornelis MC Franks PW Zhang C Hu FB Qi L Obesity genotype score and cardiovascular risk in women with type 2 diabetes mellitus Arterioscler Thromb Vasc Biol 2010 30 2 327 332 10.1161/ATVBAHA.109.196196 19910641 PMC3061473

[B61] Morrison AC Bare LA Chambless LE Ellis SG Malloy M Kane JP Pankow JS Devlin JJ Willerson JT Boerwinkle E Prediction of coronary heart disease risk using a genetic risk score: the Atherosclerosis Risk in Communities Study Am J Epidemiol 2007 166 1 28 35 10.1093/aje/kwm060 17443022

[B62] Dandona S Roberts R Creating a genetic risk score for coronary artery disease Curr Atheroscler Rep 2009 11 3 175 181 10.1007/s11883-009-0028-4 19361348

[B63] Shiffman D Rowland CM Sninsky JJ Devlin JJ Polymorphisms associated with coronary heart disease: better by the score Curr Opin Mol Ther 2006 8 6 493 499 17243484

[B64] Horne BD Anderson JL Carlquist JF Muhlestein JB Renlund DG Bair TL Pearson RR Camp NJ Generating genetic risk scores from intermediate phenotypes for use in association studies of clinically significant endpoints Ann Hum Genet 2005 69 Pt 2 176 186 10.1046/j.1469-1809.2005.00155.x 15720299 PMC4739854

[B65] Gunjaca G Boban M Pehlic M Zemunik T Budimir D Kolcic I Lauc G Rudan I Polasek O Predictive value of 8 genetic loci for serum uric acid concentration Croat Med J 2010 51 1 23 31 10.3325/cmj.2010.51.23 20162742 PMC2829178

[B66] De Jager PL Chibnik LB Cui J Reischl J Lehr S Simon KC Aubin C Bauer D Heubach JF Sandbrink R Integration of genetic risk factors into a clinical algorithm for multiple sclerosis susceptibility: a weighted genetic risk score Lancet Neurol 2009 8 12 1111 1119 10.1016/S1474-4422(09)70275-3 19879194 PMC3099419

[B67] Karlson EW Chibnik LB Kraft P Cui J Keenan BT Ding B Raychaudhuri S Klareskog L Alfredsson L Plenge RM Cumulative association of 22 genetic variants with seropositive rheumatoid arthritis risk Ann Rheum Dis 2010 10.1136/ard.2009.120170 PMC2933175 20233754

[B68] Nishiyama C Akizawa Y Nishiyama M Tokura T Kawada H Mitsuishi K Hasegawa M Ito T Nakano N Okamoto A Polymorphisms in the Fc epsilon RI beta promoter region affecting transcription activity: a possible promoter-dependent mechanism for association between Fc epsilon RI beta and atopy J Immunol 2004 173 10 6458 6464 15528387 10.4049/jimmunol.173.10.6458

[B69] Ferreira MA Zhao ZZ Thomsen SF James M Evans DM Postmus PE Kyvik KO Backer V Boomsma DI Martin NG Association and interaction analyses of eight genes under asthma linkage peaks Allergy 2009 64 11 1623 1628 10.1111/j.1398-9995.2009.02091.x 19824886

[B70] Islam T Berhane K McConnell R Gauderman WJ Avol E Peters JM Gilliland FD Glutathione-S-transferase (GST) P1, GSTM1, exercise, ozone and asthma incidence in school children Thorax 2009 64 3 197 202 10.1136/thx.2008.099366 18988661 PMC2738935

[B71] Timofeeva M Kropp S Sauter W Beckmann L Rosenberger A Illig T Jager B Mittelstrass K Dienemann H Bartsch H Genetic polymorphisms of MPO, GSTT1, GSTM1, GSTP1, EPHX1 and NQO1 as risk factors of early-onset lung cancer Int J Cancer 2010 127 7 1547 1561 20091863 10.1002/ijc.25175

[B72] Mathias RA Freidhoff LR Blumenthal MN Meyers DA Lester L King R Xu JF Solway J Barnes KC Pierce J Genome-wide linkage analyses of total serum IgE using variance components analysis in asthmatic families Genet Epidemiol 2001 20 3 340 355 10.1002/gepi.5 11255243

